# Utilisation, equity and determinants of full antenatal care in India: analysis from the National Family Health Survey 4

**DOI:** 10.1186/s12884-019-2473-6

**Published:** 2019-09-05

**Authors:** Gunjan Kumar, Tarun Shankar Choudhary, Akanksha Srivastava, Ravi Prakash Upadhyay, Sunita Taneja, Rajiv Bahl, Jose Martines, Maharaj Kishan Bhan, Nita Bhandari, Sarmila Mazumder

**Affiliations:** 1 Knowledge Integration and Translational Platform (KnIT) at the Centre for Health Research and Development, Society for Applied Studies, 45, Kalu Sarai, New Delhi, 110016 India; 2 Department of Maternal, New-born, Child and Adolescent Health, World Health Organisation, Geneva, Switzerland; 3 Centre for Intervention Science in Maternal and Child Health, Centre for International Health, University of Bergen, Bergen, Norway; 4 Indian Institute of Technology, New Delhi, India; 5 Knowledge Integration and Translational Platform (KnIT), Biotechnology Industry Research Assistance Council (BIRAC), New Delhi, India

**Keywords:** Antenatal care, India, NFHS-4

## Abstract

**Objectives:**

We examined the utilisation, equity and determinants of full antenatal care (ANC), defined as 4 or more antenatal visits, at least one tetanus toxoid (TT) injection and consumption of iron folic acid (IFA) for a minimum of 100 days, in India.

**Methods:**

We analysed a sample of 190,898 women from India’s National Family Health Survey 4. Concentration curves and concentration index were used to assess equity in full ANC utilisation. Multivariable logistic regression model was used to examine the factors associated with full ANC utilisation.

**Results:**

In India, 21% of pregnant women utilised full ANC, ranging from 2.3–65.9% across states. Overall, 51.6% had 4 or more ANC visits, 30.8% consumed IFA for atleast 100 days, and 91.1% had one or more doses of tetanus toxoid. Full ANC utilisation was inequitable across place of residence, caste and maternal education. Registration of pregnancy, utilisation of government’s Integrated Child Development Services (ICDS) and health insurance coverage were associated with higher odds of full ANC utilisation. Lower maternal education, lower wealth quintile(s), lack of father’s participation during antenatal visits, higher birth order, teenage and unintended pregnancy were associated with lower odds of full ANC utilisation.

**Conclusions:**

Full ANC utilisation in India was inadequate and inequitable. Although half of the women did not receive the minimum recommended ANC visits, the utilisation of TT immunisation was almost universal. The positive association of full ANC with ICDS utilisation and child’s father involvement may be leveraged for increasing the uptake of full ANC. Strategies to address the socio-demographic factors associated with low and inequitable utilisation of full ANC are imperative for strengthening India’s maternal health program.

**Electronic supplementary material:**

The online version of this article (10.1186/s12884-019-2473-6) contains supplementary material, which is available to authorized users.

## Background

Antenatal care (ANC) is an opportunity to promote a positive pregnancy experience and improved maternal and child survival. Care in the antenatal period is also important for supporting the long-term growth and development of the child as its part of the critical “*1000 days”* window [1–3]. It serves as a facilitating platform linking the woman and her family to the healthcare system and possibly promotes higher utilisation of essential services like breastfeeding and nutritional counselling, post-partum family planning and childhood vaccination [4, 5].

Globally, 62% of pregnant women received the WHO recommended minimum 4 antenatal visits during 2010–2016 [6]. Latest research has shown a lower still birth rate, among women with a minimum of 8 antenatal visits, based on which the minimum recommended number of antenatal contacts has now been increased from 4 to 8 [7].

In India, the proportion of pregnant women receiving the minimum 4 antenatal visits has increased from 37.0 to 51.2% during 2006–2016 [8]. This is relatively modest when compared to increase in the rate of institutional delivery which has doubled from 38.7 to 79% during the same time period, largely driven by the conditional cash transfer schemes of the government [8–10]. This differential coverage reflects a missed opportunity, as about one fourth of the maternal deaths are attributable to pre-eclampsia, eclampsia and antepartum haemorrhage, which could be identified and managed during the antenatal contacts [11].

Antenatal contacts also provide a window of opportunity to detect and possibly prevent adverse birth events. Evidence suggests that there is significant inequality in the access to essential health services, including services for pregnancy and childbirth. Disparities in utilisation of maternal care services have been reported in low and middle-income countries (LMIC) [12, 13]. In India, antenatal care is provided free of cost at public health centres. However, households incur out of pocket expenditure as private health care providers play a crucial role in providing maternity services in India [8].

A comprehensive understanding of determinants of antenatal care utilisation in India is lacking. In context of the recent focus of the Indian government on antenatal services, through Pradhan Mantri Matru Vandana Yojana (PMMVY), a conditional cash transfer scheme, understanding the factors driving the utilisation is crucial to aid the development of an informed policy. In this scheme the pregnant woman is eligible if she registers her pregnancy at the Anganwadi centre (AWC) within four months of conception, attends at least one prenatal care session and is taking Iron-folic acid tablets and TT (tetanus toxoid) injection [14].

We conducted a secondary data analysis using the recent National Family Health Survey (NFHS-4) data, with the objective of studying the coverage and equity in antenatal care utilisation at national and state level in India. We also examined the predictors of full antenatal care utilisation. The policy implications of these findings taking the new and pre-existing guidelines and national health programmes into consideration have also been discussed.

## Methods

### Data source

This analysis utilizes individual level data from the fourth round of India’s National Family Health Survey (NFHS 4), conducted during 2015–16 [8]. NFHS are a series of nationally representative, cross-sectional surveys that provides data on a range of demographic, socioeconomic, maternal and child health outcomes, reproductive health and family planning. In the fourth round, around 700,000 women, aged 15–49 years from 601,509 households were interviewed, using a two-stage stratified sampling design, with a response rate of 97%. Details of the sampling design and instruments used are available elsewhere [8].

Children recode file was used for this analysis (IAKR73FL). The recode file contains data in a standard, easy to analyse format with common variable names and coding categories for easy comparability between countries and includes summary variables and indices which are calculated post data collection [15]. Information on the utilisation of antenatal care was available for the most recent pregnancy, resulting in a live birth during the five years preceding the survey.

### Definitions and analysis

The outcome variable was “*Full antenatal care*” defined as four or more antenatal visits, at least one tetanus toxoid (TT) injection and reported consumption of iron folic acid (IFA) tablets or syrup for a minimum of 100 days [8]. Place of residence, caste, wealth quintile, access to health insurance, age at conception for current pregnancy, education, registration of pregnancy, timing of first ANC visit, utilisation of government’s Integrated child development services (ICDS), pregnancy intendedness, participation of child’s father in ANC visit and birth order of the child were used as explanatory variables. The details and sub-categories of these variables are available in Additional file 1.

The analysis was restricted to the antenatal care utilised during the last pregnancy that resulted in a live birth, in the five years preceding the survey. From the total sample of 259,627, information pertaining to last live birth was available for 190,898 births. The proportion of pregnant women who utilised full ANC was estimated at national and sub-national level (states and union territories). There are 36 states/union territories in India. We used binary logistic regression to examine the association between the explanatory variables and full ANC care.

The variables with a *p*-value of < 0.20 in univariable analysis and variables of known clinical or contextual importance were included in the multivariable model [16]. A *p*-value of < 0.05 was considered as statistically significant for all analyses. We used concentration curves and concentration index to examine the extent of wealth-based inequality in utilisation of full ANC in India and states/union territories. They capture the extent to which health differs across individuals ranked by some indicator of socioeconomic status [17]. In this analysis, concentration curve plots the cumulative proportion of full antenatal care utilisation against the cumulative proportion of the population ranked by wealth index. If the concentration curve lies above the 45° line (line of equality), it means its utilisation is more concentrated amongst those with lower incomes and vice versa. The concentration index is defined as twice the area between the concentration curve, and the line of equality. It ranges from − 1 to + 1 and the concentration index of zero implies no socioeconomic-related inequality.

Analysis was done using STATA© 15.1 (StataCorp LLC, College Station, TX, USA). Stata’s survey command (*svyset*) was used to adjust for sampling weight, clustering and stratification in the sampling design. User written Stata package “*conindex*” was used for calculating the concentration index. The guidelines for data use as required by the DHS program were strictly followed [18].

## Results

We report the findings for 190,898 women, who had at least one live birth in the five years preceding the survey. Background characteristics of these women are shown in Table 1.

**Table 1:** Background characteristics of women who gave birth in last five years, NFHS-4 (2015–16)

Characteristics	Categories	Frequency (weighted %)
Wealth quintiles	Lowest	46,782 (23.4)
	Second	43,739 (21.5)
	Middle	38,393 (19.9)
	Fourth	33,212 (19)
	Highest	28,772 (16.6)
Social caste	^a^Others	34,705 (21.1)
	Scheduled caste	35,170 (22)
	Scheduled tribe	37,889 (10.7)
	Other Backward Categories	74,060 (45.3)
Place of residence	Urban	47,833 (29.7)
	Rural	143,065 (70.3)
Health insurance cover	No	163,367 (84.7)
	Yes	27,531 (15.3)
Maternal age at conception (in completed years	less than 19 years	16,225 (9.5)
	19–30 years	150,683 (80)
	More than 30 years	23,990 (10.5)
Maternal education	No or less than one year of formal education	55,165 (27.6)
	Up to Primary (1–5 years of schooling)	26,712 (13.5)
	Secondary (6–12 years of schooling)	88,871 (46.9)
	Higher Education (more than 12 years of schooling)	20,150 (12)
Birth order	First	61,807 (33.6)
	Second	62,484 (34.5)
	Third	33,064 (16.6)
	Four or higher	33,543 (15.4)
Intended pregnancy	Yes	173,407 (90.8)
	Did not wanted/wanted later	17,390 (9.2)
Previous miscarriage/abortion/still birth	Yes	19,671 (10.3)
	No	171,227 (89.7)
Pregnancy registered	Yes	160,769 (85.4)
	No	30,028 (14.6)
Timing of first ANC visit	1st trimester	43,776 (28.4)
	2nd trimester	101,278 (63.3)
	3rd trimester	11,701 (8.04)
Presence of child’s father at any ANC visit	No	31,291 (17.7)
	Yes	125,965 (82.3)
Received government’s Integrated Child Development Service (ICDS) benefits	Yes	105,980 (55.4)
	No	84,824 (44.6)

^a^Others include people not belonging to SC, ST or OBC

### Full ANC utilisation

**Fig. 1:**
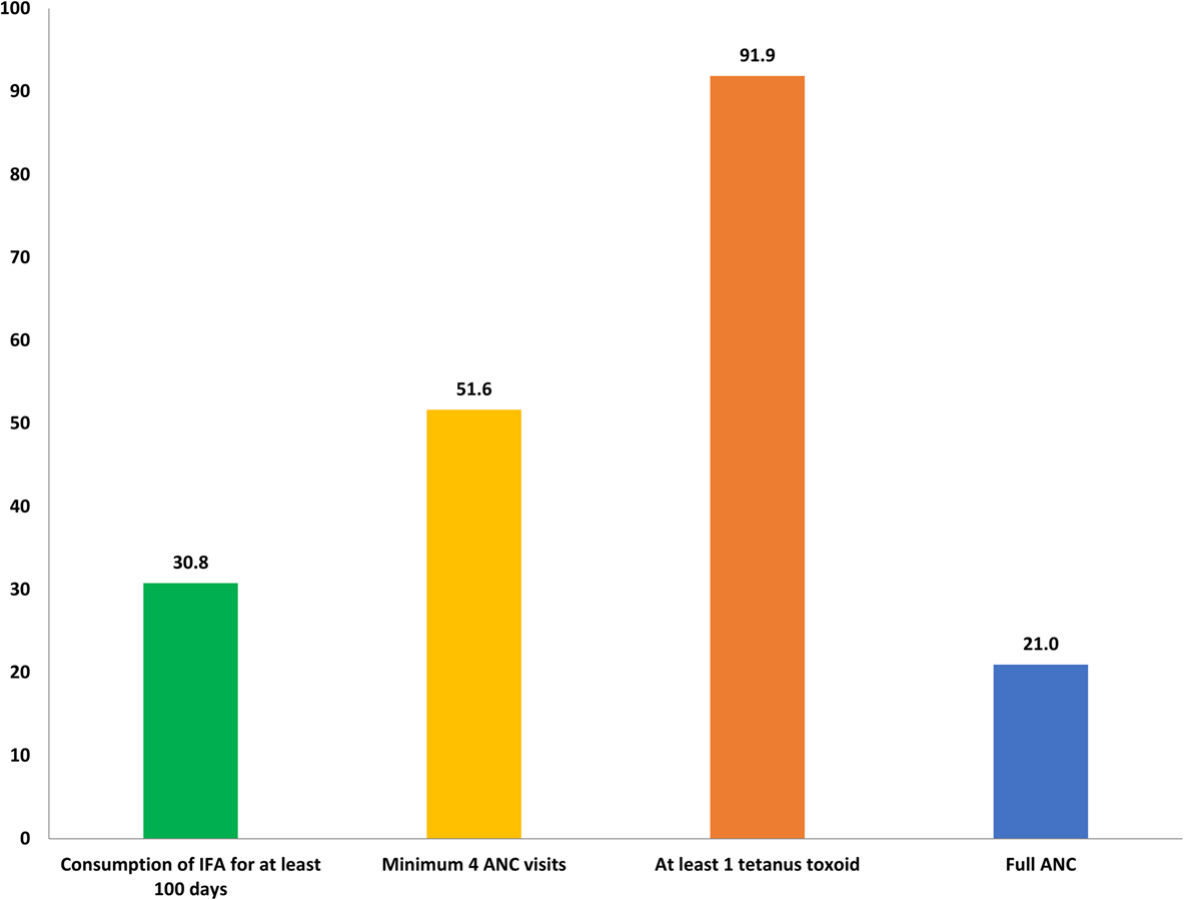
Utilisation (%) of full antenatal care and different components in India, NFHS-4 (2015–16)

**Fig. 2:**
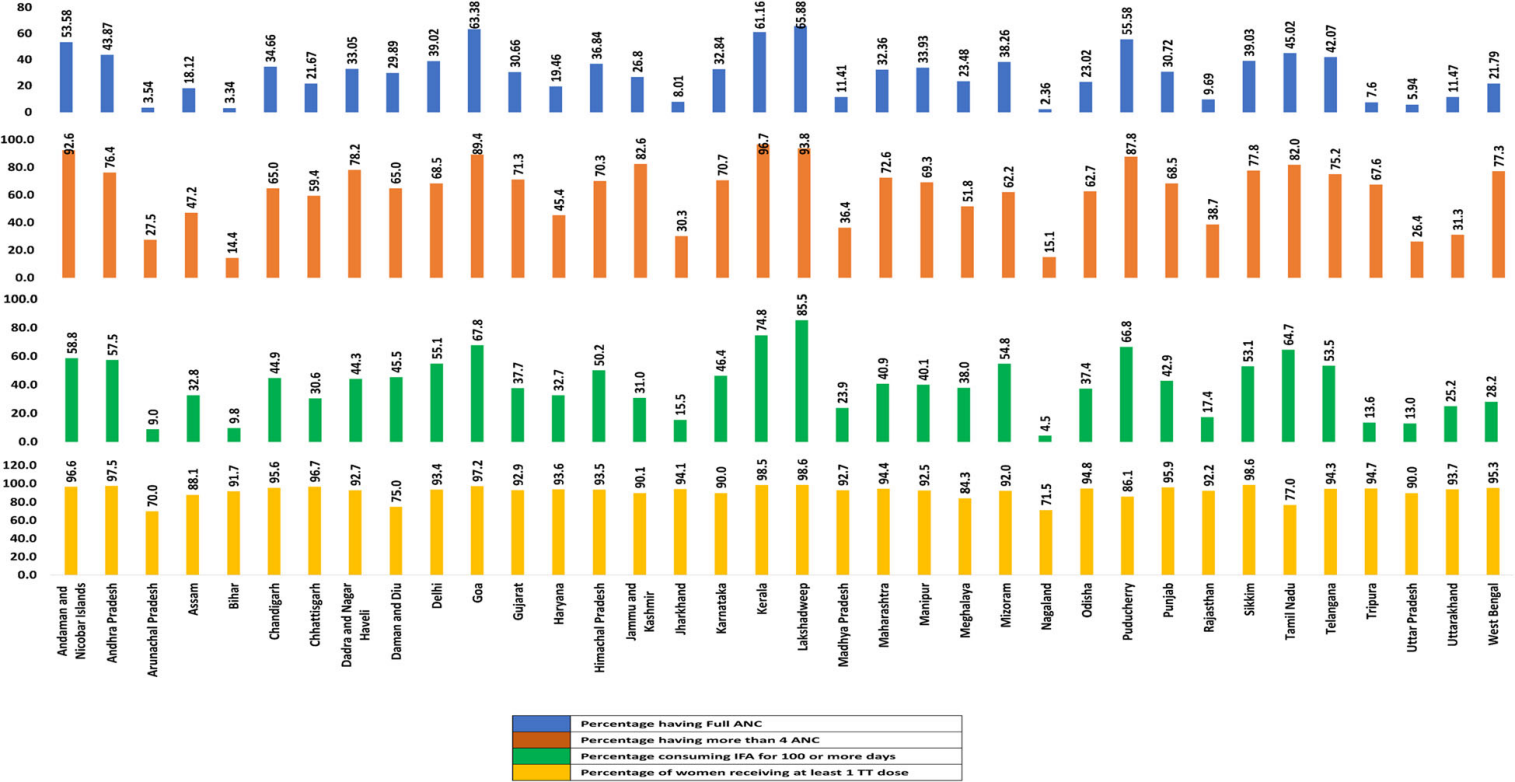
State-wise utilisation (%) of full antenatal care and its components in India, NFHS-4 (2015–16)

Twenty One percent of pregnant women utilised full ANC during their last pregnancy ranging from 2.3–65.9% within states (median 30.7, IQR 14.8–39.0). The proportion of women who had a minimum of 4 ANC visits was 51.6%, ranging from 14.4–96.7% within states, IFA was consumed for a minimum of 100 days by 30.8% of women, ranging from 4.5–85.5% within states. At least one dose of tetanus toxoid was received by 91.1% of women ranging from 70 to 98.6% within states (Figs. 1 and 2). The median number of antenatal visits in the country was 4 (IQR 2–7).

### Inequity in full ANC utilisation

**Fig. 3:**
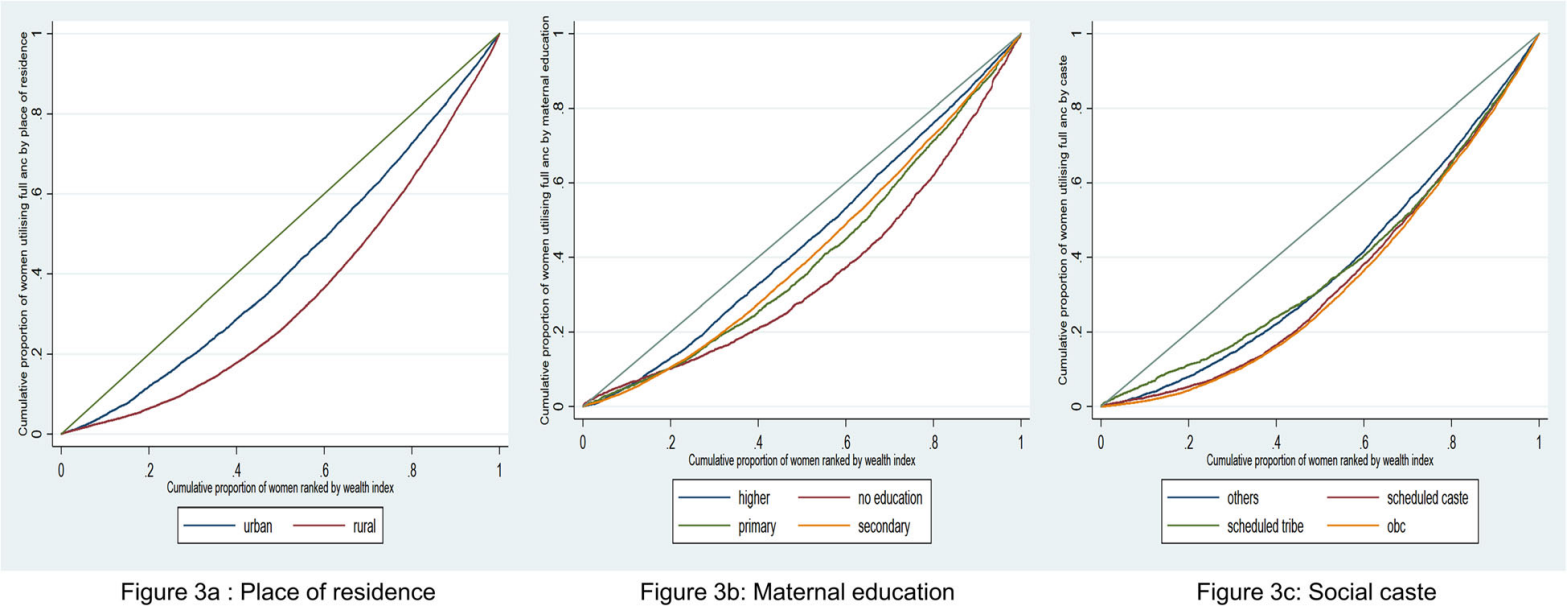
Concentration curves for full antenatal care utilisation across place of residence (**a**), maternal education (**b**), social caste (**c**)

**Fig. 4:**
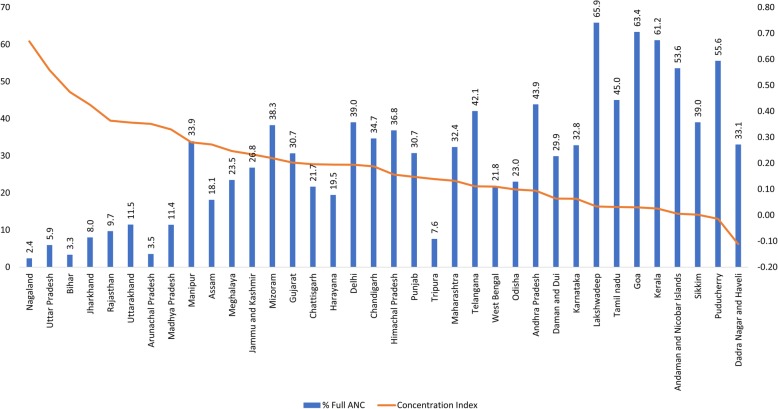
State-wise inequity in full antenatal care utilisation in India, NFHS-4 (2015–16)

The inequity in full ANC utilisation was higher in rural areas, among women with no education and those belong to socially disadvantaged groups (SC/ST/OBC) (Fig. 3a-c). The utilisation of full ANC, was highly inequitable with a concentration index of 0.31, ranging from − 0.11 to 0.67 within states. In general, the states with lower utilisation of full ANC had higher level of inequity in utilisation (Fig. 4).

### Determinants of full ANC

Results from the multivariable logistic regression model are presented in Table 2. The model was also adjusted for respondent’s state of residence. We observed a gradient in full ANC utilisation across the wealth quintiles, the highest utilisation was among the richest and vice-versa. Women residing in rural areas had lower odds (adjusted odds ratio, AOR 0.90; 95% CI 0.84–0.96) of full ANC utilisation. Odds of full ANC utilisation was higher among women covered by health insurance (AOR 1.18; 95% CI 1.11–1.26).

**Table 2:** Determinants of full antenatal care utilisation in India, NFHS-4 (2015–16)^d^

Determinants (Weighted N)^a^	Proportion of women with Full ANC % (weighted n)^b^	Unadjusted odds ratio (95% CI)	Adjusted odds ratio (95% CI)
Socioeconomic indicators			
Wealth Quintiles			
Lowest (46782)	3126 (6.7)	0.12(0.11–0.13)	0.47 (0.42–0.52)
Second (43739)	5531 (14.2)	0.27(0.25–0.29)	0.56 (0.52–0.62)
Middle (38393)	7567 (22.6)	0.48(0.45–0.51)	0.66 (0.61–0.71)
Fourth (33212)	8675 (29.2)	0.67(0.63–0.71)	0.75 (0.70–0.81)
Highest (28772)	9967 (38.1)	(Reference)	(Reference)
Social caste			
^c^Others (34,705)	8373 (26.2)	(Reference)	(Reference)
Scheduled caste (35,170)	5686 (19.3)	0.67(0.62–0.73)	0.95 (0.87–1.03)
Scheduled tribe (37,889)	6288 (16.3)	0.55(0.51–0.60)	0.92 (0.84–1.01)
Other Backward Categories (74,060)	13,037 (20.7)	0.74(0.69–0.78)	0.94 (0.88–1.00)
Place of Residence			
Urban (47,833)	13,074 (31.1)	(Reference)	(Reference)
Rural (143,065)	21,792 (16.7)	0.44(0.42–0.47)	0.90 (0.84–0.96)
Health insurance cover			
No (163,367)	27,733 (18.8)	(Reference)	(Reference)
Yes (27,531)	7133 (32.8)	2.11(2.00–2.22)	1.18 (1.11–1.26)
Maternal indicators			
Maternal age at conception (years)			
Less than 19 years (16,225)	2594 (18.7)	0.83(0.77–0.88)	0.86 (0.79–0.93)
19–30 years (150,683)	28,367 (21.7)	(Reference)	(Reference)
More than 30 years (23,990)	3905 (17.3)	0.75(0.71–0.80)	1.16 (1.07–1.27)
Maternal Education			
No or less than one year of formal education (55,165)	4103 (8.2)	0.14(0.13–0.15)	0.56 (0.51–0.62)
Up to Primary (1–5 years of schooling) (26,712)	3401 (14.8)	0.27(0.25–0.29)	0.64 (0.58–0.70)
Secondary (6–12 years of schooling) (88,871)	20,117 (25.6)	0.54(0.50–0.57)	0.75 (0.70–0.80)
Higher education (20150)	7245 (39.1)	(Reference)	(Reference)
Birth order			
First (61807)	14,756 (26.4)	(Reference)	(Reference)
Second (62484)	12,985 (24.2)	0.89(0.85–0.93)	0.93 (0.88–0.98)
Third (33064)	4618 (15.7)	0.52(0.49–0.55)	0.84 (0.78–0.90)
4 or more (33543)	2507 (7.2)	0.22(0.20–0.23)	0.68 (0.62–0.75)
Intended pregnancy			
Yes (173,407)	32,904 (21.8)	(Reference)	(Reference)
Did not want/later (17,390)	1962 (12.5)	0.51(0.47–0.50)	0.83 (0.76–0.92)
Previous miscarriage/abortion/still birth			
Yes (19,671)	3975 (22.6)	1.12 (1.04–1.18)	1.13 (1.06–1.21)
No (171,227)	30,891 (20.8)	(Reference)	(Reference)
Care during antenatal period			
Pregnancy registered			
Yes (160,769)	32,675 (23.0)	(Reference)	(Reference)
No (30,028)	2191 (8.8)	0.32(0.30–0.35)	0.78 (0.71–0.85)
Timing of first ANC visit			
1st trimester (43,776)	14,322 (36.0)	(Reference)	(Reference)
2nd trimester (101,278)	19,016 (21.3)	0.48(0.46–0.51)	0.72 (0.69–0.76)
3rd trimester (11,701)	1514 (16.9)	0.36(0.33–0.40)	0.47 (0.42–0.53)
Presence of child’s father at any ANC visit			
No (31,291)	4538 (15.7)	0.50 (0.47–0.53)	0.72 (0.68–0.77)
Yes (125,965)	30,328 (27.1)	(Reference)	(Reference)
Received ICDS benefits			
Yes (105,980)	21,252 (22.0)	(Reference)	(Reference)
No (84,824)	13,614 (19.6)	0.86(0.83–0.90)	0.93 (0.88–0.98)

^a^N = row total, ^b^n = weighted frequency, ^c^others include people not belonging to SC, ST or OBC

^d^Model adjusted for state of residence

Maternal age below 19 years at conception, had lower odds (AOR 0.86; 95% CI 0.79–0.93) of full ANC utilisation. A gradient in full ANC utilisation was also observed across the levels of education, with lowest utilisation among those with no formal education. The odds of full ANC decreased with increasing birth order. Unintended pregnancy was associated with a lower odds of full ANC utilisation (AOR 0.83; 95% CI 0.76–0.92).

Unregistered pregnancy was associated with lower odds of full ANC utilisation (AOR 0.78; 95% CI 0.71–0.85). Women who had their first ANC visit in the second or third trimester had lower odds of full ANC utilisation. Lack of child’s father participation during the ANC visits was associated with lower odds of full ANC utilisation, (AOR 0.72; 95% CI 0.68–0.77). Women who had not received ICDS benefits had lower odds of full ANC utilisation (AOR 0.93; 95% CI 0.88–0.98). Caste was not associated with full ANC utilisation.

## Discussion

The findings of this analysis are of concern as despite efforts of the Indian government during the last two decades, only one fifth of pregnant women utilised full ANC. Half of the women did not receive the minimum recommended 4 ANC visits, which are a conservative expectation when compared to the recent recommendations of the World Health Organisation (WHO), a minimum of 8 visits. In 17 out of 36 states/UTs, less than 30% of the pregnant women received full ANC. Inequity in full ANC utilisation was higher in states with low rates of full ANC coverage.

The proportion of women with 4 or more ANC visits is considerably lower than the global average of 61.8% and implementing the recent WHO recommendation of a minimum of 8 ANC visits will be a major challenge for the national programme in India [6]. The number of ANC visits may also be critical to the delivery of other components of ANC and to provide adequate follow-up of pregnant women closer to delivery. The high proportion of mothers with at least one tetanus toxoid immunisation can be achieved even in a single visit during any trimester. In contrast, 100 days of IFA consumption is possible only if multiple visits are made as currently the supplies are given for 1 month at each visit and lower number of visits may be a reason for low utilisation for100 days of IFA.

Registration of pregnancy, utilisation of benefits from the government’s ICDS program and having health insurance were associated with higher odds of full ANC utilisation. Lower maternal education, lower wealth quintile(s), higher birth order, father not accompanying for the ANC visit, teenage pregnancy and unintended pregnancy were associated with lower odds of full ANC utilisation.

Economic status and maternal education were highly associated with utilisation of ANC, which corroborates with previous literature [19–21]. Younger maternal age at conception is also common among these women. In LMICs including South East Asia, women from the highest wealth quintile have higher financial and social access to health care services in general, which may lead to higher utilisation of full ANC as seen in the current analysis [6].

Unintended pregnancy, which may be due to lack of awareness or, a reflection of inadequate or inaccessible family planning services has been associated with lower ANC utilisation previously and our findings also suggest the same [22, 23].

Lower ANC utilisation among women of higher parity can be due to increased confidence from previous pregnancy and childbirth experience, constraints of time and resources, poor prior experience with the health system and financial barriers to ANC utilisation [19].

Equity in states with low ANC utilisation is expected to be lower as those who are better-off are more likely to utilise the services. This finding is critical to account for while redesigning of strategies for increasing ANC utilisation at national and subnational level. As India prepares for achieving universal health coverage, equity in utilisation of essential maternal healthcare services like ANC should be ensured.

Child’s father presence during any ANC visit was associated with higher utilisation of full ANC, this is of special interest in the context of a patriarchal society like India. Child’s father presence during ANC visit, may reflect greater spousal care and support, joint decision making and a more caring environment at home [24, 25]. We found a positive association between health insurance coverage and full ANC utilisation in the adjusted analysis. However, only 20% women were covered by health insurance or health schemes and majority of these were beneficiaries of central or state government health insurance schemes [8]. This finding is relevant to the current health policy as majority of the private insurance players and even the recently launched National Health Protection Scheme (“Ayushman Bharat”) do not cover ANC services [26]. Higher utilisation of government’s ICDS services, implemented through Anganwadis which are the focal point of community outreach sessions for antenatal care, health education and nutrition support during pregnancy and lactation was associated with higher odds of full ANC as it acts as a bridging platform for different government schemes like Janani Suraksha Yojna (JSY) and Pradhan Mantri Matru Vandana Yojna (PMMVY) [14, 27].

### Strengths and limitations

The findings from this analysis should be interpreted considering the following strengths. Our analysis uses data from the most recent survey with a large, nationally representative sample, and is based on a more comprehensive indicator i.e. *full ANC* (minimum of 4 ANC visits, consumption of 100 or more days of IFA and at least 1 dose of tetanus toxoid), as against the total number of ANC visits used in previous studies. Some limitations are as follows. Lack of information on pregnancies which did not result in a live birth i.e. abortion, miscarriage or still birth restricts us from commenting on the utilisation of ANC in this subset with adverse outcomes. The responses pertaining to the individual components of full ANC, were self-reported and therefore prone to recall bias, although the subgroup analysis restricted to births in the one year preceding the survey yielded similar results. We are unable to comment on some factors like care seeking behaviour, health literacy, distance from the health care facility, provider discrimination and other system side factors for which information was not collected in NFHS-4.

## Conclusions

Full ANC utilisation in India was inadequate and inequitable. Although half of the women did not receive the minimum number of recommended ANC visits, the utilisation of TT immunisation was almost universal. In a country like India, where still birth, prematurity, Intrauterine growth restriction (IUGR) and maternal mortality are still high, the recent WHO recommendations of 8 ANC visits, emphasises the need to provide more number of ANC visits and (should force us to recognise the need) intensive care to pregnant women [28–30]. Taking into consideration the factors associated with low and inequitable utilisation, it is imperative to design strategies, in order the achieve the WHO recommendations at the earliest.

Counselling of young married couples may create a channel for greater information flow, for planned parenthood. Broadcasting important messages related to antenatal care at relevant health and educational institutes and through mass media, may help in increasing awareness at the population level. Future surveys should capture details of antenatal care with higher granularity to understand the quality of care received at each visit. The states with low full ANC utilisation should give greater priority to improving ANC services and using the opportunities opening in the country [14]. Coordinated effort is needed from the different sections of the government involved in delivering maternal care during pregnancy. We suggest, that the newly launched Ayushman Bharat scheme, should also prioritize covering outpatient-based antenatal care.

## Additional file

Additional file 1: Details of the variables used for analysis. (DOCX 31 kb)

## Data Availability

Data is available for academic research purposes from the following link after registration as a DHS data user: https://www.dhsprogram.com/data/dataset/India_Standard-DHS_2015.cfm?flag=0.

## Abbreviations

95% CI: 95% Confidence Interval
ANC: Antenatal Care
AOR: Adjusted Odds Ratio
AWC: Anganwadi Centre
ICDS: Integrated Child Development Scheme
IFA: Iron Folic Acid
IQR: Inter Quartile Range
IUGR: Intrauterine Growth Retardation
JSY: Janani Suraksha Yojana
LMIC: Low- and Middle-Income Countries
NFHS: National Family Health Survey
PMMVY: Pradhan Mantri Matru Vandana Yojana
TT: Tetanus Toxoid
WHO: World Health Organisation
