# Supplementary material for: Utilisation, equity and determinants of full antenatal care in India: analysis from the National Family Health Survey 4
Source: BMC Pregnancy Childbirth. 2019 Sep 5;19:327. doi: 10.1186/s12884-019-2473-6 (PMC6727513; doi:10.1186/s12884-019-2473-6)
Supplement: Supplementary file 1 — Details of the variables used for analysis. (DOCX 31 kb) [file 12884_2019_2473_MOESM1_ESM.docx]

**Details of the variables used for analysis**

| Variable | Categories |
| --- | --- |
| **Socioeconomic determinants** | |
| Place of residence | Urban |
|  | Rural |
| Wealth quintile | Lowest |
|  | Second |
|  | Middle |
|  | Fourth |
|  | Highest |
| Social caste | Scheduled Caste (SC)* |
|  | Scheduled Tribe (ST)* |
|  | Other Backward Caste (OBC)^#^ |
|  | Others (do not belong to Scheduled caste/Tribe and other backward castes) |
| Health insurance | No (No Health Insurance cover) |
|  | Yes (Has Health Insurance cover) |
| Maternal education | Higher/Secondary (> 12 years of schooling) |
|  | Secondary (6-12 years of schooling) |
|  | Up to Primary (1-5 years of schooling) |
|  | No education (No or < 1 year of formal education) |
| **Maternal characteristics** | |
| Intended to get pregnant | Yes (Wanted to get pregnant) |
|  | No/ Later (Did not want to get pregnant/ wanted pregnancy at a later time) |
| Maternal age at conception (In complete years) | < 19 years |
|  | - 1. years |
|  | > 30 years |
| Birth order | First |
|  | Second |
|  | Third |
|  | Fourth or more |
| **Care during antenatal period** | |
| Pregnancy registered (registration of pregnant women with a formal health system, as reported by women) | Yes |
|  | No |
| Timing of first ANC visit | 1st Trimester (1-13 weeks) |
|  | 2nd trimester (14-26 weeks) |
|  | 3rd trimester (27-40 weeks) |
| Presence of child’s father at any ANC visit | No (Child’s father has never accompanied his wife at any of the ANC visits) |
|  | Yes (Child’s father has accompanied her wife for at least one ANC visit) |
| Received any Integrated Child Development Services (ICDS) benefits (supplementary food, health check-up, nutrition counselling, referral under Integrated Child development services scheme) | Yes (Received any of the services provided under ICDS for pregnant women) |
|  | No (Did not receive any of the services provided under ICDS for pregnant women) |

*The Scheduled Castes (SCs) and Scheduled Tribes (STs) are officially designated groups of historically disadvantaged people in India.

#Other Backward Caste (OBC) is a collective term used by the Government of India to classify castes which are educationally or socially disadvantaged.
